# Off-label use of atypical antipsychotics- Where are we?

**DOI:** 10.1192/j.eurpsy.2023.681

**Published:** 2023-07-19

**Authors:** R. P. Vaz, J. Martins, A. L. Costa, J. Brás, R. Sousa, E. Almeida, J. Abreu, N. Castro, R. Andrade, N. Gil

**Affiliations:** Departamento de Psiquiatria e Saúde Mental, Centro Hospitalar Tondela Viseu, Viseu, Portugal

## Abstract

**Introduction:**

Nowadays, In the exercise of psychiatric clinical activity, the prescription of atypical antipsychotics is a widespread practice.

However, despite the approval in the treatment of psychoses and bipolar affective disorder, where its effectiveness is clearly demonstrated, these drugs are off-label prescribed in most of the clinical situations.

**Objectives:**

This work aims to clarify which atypical antipsychotics are most frequent prescribed and the clinical conditions where their off-label prescription is more common.

**Methods:**

Bibliographic research in the Pubmed® database using the terms “atypical antipsychotics and off-label use”

**Results:**

According to the scientific literature consulted, the off-label prescription of atypical antipsychotics may represent about 70% of the total prescription of these psychotropic drugs.

Risperidone, olanzapine, quetiapine and aripiprazole are the most off-label prescribed among the atypical antipsychotics.

The psychiatric conditions where atypical antipsychotics are most often off-label prescribed are addictive disorders, anxiety disorders, post-traumatic stress disorder, personality disorders, eating disorders, insomnia and dementia, where therapeutic benefits are demonstrated when carefully selected.

**Conclusions:**

The off-label prescription can be interpreted from two points of view. On the one hand, it can guide innovation in clinical practice and improve symptoms in patients who do not respond to standard treatments. On the other hand, it may be associated with negative consequences due to the lack of data on safety and efficacy in these situations.

Despite widespread prescribing of atypical antipsychotics, there is no evidence-based recommendation beyond psychoses and bipolar affective disorder.

Thus, when prescribed, we must proceed with careful monitoring and consider the risks and benefits in relation to off-label prescription.

**Disclosure of Interest:**

None Declared

